# LncRNA FEZF1-AS1 regulates the gene expression and alternative splicing associated with osteosarcoma

**DOI:** 10.1186/s13018-025-06357-z

**Published:** 2025-12-03

**Authors:** Yanlong Han, Ziang Ye, Li Wang

**Affiliations:** Department of Bone and Joint Surgery Center, People’s Hospital of Xinjiang Uygur Autonomous Region, Urumqi, 830001 China

**Keywords:** LncRNA FEZF1-AS1, Osteosarcoma, RNA Sequencing, Alternative Splicing

## Abstract

**Background:**

Osteosarcoma (OS) faces a high metastatic burden, limited effective treatments, and a lack of specific therapeutic targets.

**Methods:**

In this prospective study, we established a human osteosarcoma cell (HOS) model overexpressing lncRNA FEZF1-AS1 (OE FEZF1-AS1), and subsequently obtained FEZF1-AS1-affected transcriptome data using high-throughput RNA sequencing (RNA-seq).

**Results:**

OE FEZF1-AS1 promoted HOS cell proliferation and invasion, upregulated 125 genes, suppressed 48 genes, and impacted 1156 alternative splicing events (ASEs), including pathways like C-type lectin receptor signaling and *Yersinia pestis* infection.

**Conclusions:**

LncRNA FEZF1-AS1 plays a pivotal role in promoting the proliferation and invasion of HOS cells. Moreover, our study provides a comprehensive gene screening approach for identifying potential targets of FEZF1-AS1, emphasizing their regulatory mechanisms and ASEs in OS.

**Supplementary Information:**

The online version contains supplementary material available at 10.1186/s13018-025-06357-z.

## Introduction

Osteosarcoma (OS) is the most prevalent primary skeletal malignancy and the second leading cause of cancer death in adolescents [1], with a high propensity for local invasion and metastasis. The clinical manifestations of OS include bone pain and swelling [2]. Adjuvant chemotherapy (AC) following limb-sparing surgery with endoprosthesis is the gold standard treatment for OS [3]. Despite significant improvement in the prognosis of OS patients achieved through combination chemotherapy and surgical interventions, the prognosis for patients with metastatic or recurrent OS remains unsatisfactory. Currently, the challenges associated with OS include a high rate of metastasis, a paucity of effective therapeutic agents, substantial heterogeneity, and a lack of specific targets. Therefore, there is an urgent need to explore safe, effective, and well-tolerated therapeutics to cure or delay the progression of OS.

Long non-coding RNAs (lncRNAs) refer to a class of RNA molecules that are more than 200 nucleotides in length, which do not have protein-coding ability but rather exert diverse biological functions by regulating gene expressions and functions at transcriptional, translational, and post-translational levels [4]. It has been documented that lncRNAs exert a pivotal role in various cellular processes, such as cell cycle, differentiation, and metabolism [5]. On the other hand, mounting evidence has highlighted the critical role of lncRNAs in the regulation of diverse polygenic human diseases, particularly within the musculoskeletal system, encompassing tendon injuries [6], tendon degeneration [7], osteoarthritis [8], rheumatoid arthritis [9], and osteoporosis [10, 11]. Numerous studies have underscored the critical function of lncRNAs in the pathogenesis of OS, as well as their involvement in regulating pivotal processes such as onset, growth, invasion, systemic dissemination, and drug resistance [12], implying that we can use lncRNAs as future diagnostic and prognostic clinical indicators as well as therapeutic targets for OS patients.

LncRNA FEZ family Zinc Finger 1-Antisense RNA 1 (FEZF1-AS1), as a newly discovered lncRNA, is mapped to chromosome 7q31.32, with a length of 2653 bp [13]. FEZF1-AS1 has been reported to regulate tumor progression in various cancers, including hepatocellular carcinoma [14], gastric cancer [15] and other human malignancies, and is associated with poor prognosis [16]. As an oncogene, it plays a crucial role in the proliferation [17], migration [18], invasion [19] and Warburg effect [20] of various tumor cells. According to previous studies, FEZF1-AS1 was significantly overexpressed in OS tissues and cells, and silencing of FEZF1-AS1 resulted in the inhibition of proliferation, migration, and invasion in HOS cells [21]. Furthermore, FEZF1-AS1 has been shown to regulate OS progression by sponging miR-144 and miR-4443 [21, 22]. The aforementioned studies suggest that FEZF1-AS1 is mainly involved in tumorigenesis and progression through competing endogenous RNA (ceRNA), which sponges tumor-suppressive microRNA (miRNA) and recruiting mechanism [23]. However, the existence of other potential molecular mechanisms involving FEZF1-AS1 in OS, particularly in the context of transcriptional regulation, remains to be elucidated.

Alternative splicing (AS) tightly addresses many critical aspects of cellular function, from cell proliferation and differentiation to tissue development and response to xenobiotics [24]. In this process, non-coding sequences of pre-mRNA are removed and protein-coding segments are assembled in diverse combinations, ultimately giving rise to proteins with distinct or even opposing functions [25, 26]. Three fundamental aspects of oncogenesis: cell cycle [27], DNA damage response [28] and apoptosis [29] have been shown to be largely regulated by AS. Among these, aberrant regulation of AS has been shown to be associated with the development and prognosis of OS [30, 31]. Dai et al. conducted a comprehensive screening of 63 ASEs associated with OS with high credibility and dominance [32]. A large body of literature suggests that lncRNAs regulate AS to alter the activity of a chromatin remodeler and promote metastatic behaviors in various cancer cells [24, 33]. However, the specific selective AS regulated by lncRNA FEZF1-AS1 and its mechanism are still unclear and need to be further investigated.

Thus, in the present study we established a OE FEZF1-AS1 cell model in HOS cells and analyzed the FEZF1-AS1 affected transcriptome data (RNA-seq) by high-throughput sequencing. Our results revealed the regulatory changes of relevant genes and AS in OS after OE FEZF1-AS1, allowing for a more comprehensive understanding of the causes of OS development and providing new research directions for OS immunotherapy or targeted therapy.

## Materials and methods

### Cell culture and transfections

The human OS cell lines (CL-0360; Procell Life Science & Technology Co., Ltd., China) were cultured at 37 °C with 5% CO_2_ in MEM (PM150410; Procell Life Science & Technology Co., Ltd., China) with 10% fetal bovine serum (FBS; 10099–141, Gibco, China), 100 μg/mL streptomycin, and 100 U/mL penicillin. The pcDNA3.1-FEZF1-AS1 plasmid (NR_036484.1; Youbio Biotech, Changsha, China) was transfected into HOS cells using Lipofectamine 3000 (L3000015; Invitrogen, Carlsbad, CA, USA) according to the manufacturer's protocol. After 48 h, the transfected cells were harvested for RT-qPCR analysis.

### Cell proliferation assay and invasion assay

The cell proliferation assay was conducted using a Cell Counting Kit-8 (CCK-8; HYK0301, MCE, Shanghai, China). Briefly, HOS cells were seeded at 35,000 cells/well in 24-well culture plates. The cells in the control group and the experimental group were treated accordingly, and vials without cells were used as blank controls. Following culture for 24 h at 37 °C and 5% CO_2_, 40 μL of CCK-8 solution was added to the culture medium and incubated for an additional 3 h at 37 °C. The optical density (OD) of the cells was then measured using a microplate reader (ELX800; Biotek, USA) at an absorbance wavelength of 450 nm. The cell proliferation rate was calculated using the following formula:

$$ \begin{aligned} & Proliferation~\,rate \\ = & \, \frac{{Experimental\,~OD~\,value - Blank\,~OD~\,value}}{{{\text{Control}}\,{\text{OD~}}\,{\text{value}} - {\text{Blank}}\,{\text{~OD~}}\,{\text{value}}}} \times {\text{1}}00\% \\ \end{aligned} $$

In vitro invasion assays were performed using transwell chambers (3422; Corning, USA). The transwell chambers with an 8 µm filter and precoated with a thin layer of Matrigel (356234; BD Biosciences, USA), diluted for 1:8 using serum-free medium. A total of 100 µL of Matrigel diluted in chambers was incubated for 1 h at 37 °C and 5% CO_2_ and removed unsolidified supernatant. The inserts were then filled with 5 × 10^4^ to 10^5^ cells in 0.2 mL serum-free medium. The transwell chambers were inserted into the medium containing 600 uL of 10% FBS (10099–141; Gibco, USA) in the lower chamber. The chambers were then incubated for 24–48 h at 37 °C and 5% CO2. The cells on the upper surface were then removed using a cotton swab. The total number of cells that had invaded the lower chamber was fixed using 4% paraformaldehyde (P0099; Beyotime, China) for 20 min. Then, the cells were stained with 0.1% crystal violet (C0121; Beyotime, China). The invasion cells were then observed and enumerated under an inverted microscope (MF52-N; Mshot, China) at 200 × magnification.

### RNA extraction and sequencing

Total RNA was subjected to treatment with RQ1 DNase (Promega) to eliminate DNA contamination. The quality and quantity of the purified RNA were determined by measuring the absorbance at 260 nm/280 nm (A260/A280) using a SmartSpec Plus spectrophotometer (Bio-Rad). RNA integrity was further verified by 1.5% agarose gel electrophoresis. For each sample, total RNA was utilized for directional RNA-seq library preparation by RNA-seq Library Prep Kit for Illumina (N605) for RNA-seq library preparation. mRNAs were captured by VAHTS mRNA Capture Beads (Vazyme, N401) or ribosomal RNAs were depleted with rRNA depletion kit (Vazyme, N406-01). Fragmented mRNAs were converted into double-strand cDNA. Following end repair and A tailing, the DNAs were ligated to VAHTS RNA Multiplex Oligos Set 1 for Illumina (N323); the ligated products were amplified, purified, quantified, and stored at − 80 °C prior to sequencing. The strand marked with dUTP (the second cDNA strand) was not amplified, allowing for strand-specific sequencing.

For high-throughput sequencing, the libraries were prepared according to the manufacturer's instructions and subsequently applied to Illumina Novaseq 6000 system for 150 nt paired-end sequencing. Raw reads containing more than 2-N bases were first discarded. Then adaptors and lowquality bases were trimmed from raw sequencing reads using FASTX-Toolkit (Version 0.0.13). The short reads less than 16nt were also dropped. After that, clean reads were aligned to the GRCh38 genome by HISAT2 (Kim, Langmead et al. 2015) allowing 4 mismatches. Uniquely mapped reads were used for gene reads number counting and FPKM calculation (fragments per kilobase of transcript per million fragments mapped)(Trapnell, Williams et al. 2010).

### Differentially expressed genes (DEGs) analysis

The R Bioconductor package DESeq2 (Love, Huber et al. 2014) was utilized to screen out the DEGs. The cut-off criteria for identifying DEGs were set as *p* < 0.05 and fold change > 1.5 or < 0.67.

### Functional enrichment analysis

To categorize the DEGs according to their function, Gene Ontology (GO) terms and KEGG pathways were identified using KOBAS 2.0 server (Xie, Mao et al. 2011). The hypergeometric test and the Benjamini–Hochberg false discovery rate (FDR) controlling procedure were used to define the enrichment of each term. Genes with an adjusted p-value (false discovery rate, FDR) < 0.05 and |log₂(fold change)|> 1 were considered statistically significant.

### Alternative splicing analysis

The ASEs and regulated alternative splicing events (RASEs) between the samples were defined and quantified by using the ABLas pipeline as described previously (Jin, Li et al. 2017, Xia, Chen et al. 2017). In brief, ABLas detection of ten types of ASEs was based on the splice junction reads, including exon skipping (ES), alternative 5' splice site (A5SS), alternative 3'splice site (A3SS), mutually exclusive exons (MXE), mutually exclusive 5'UTRs (5pMXE), mutually exclusive 3'UTRs (3pMXE), cassette exon, A3SS&ES and A5SS&ES.

### Statistical analysis

All Data was expressed as means ± standard deviations (SD) and analyzed by using SPSS 23.0 software (IBM Corporation, Armonk, NY, USA). GraphPad Prism 8.0 (GraphPad Software, La Jolla, CA, USA) was used to generate graphs. The test level of homogeneity of variance was 0.05. When the data met the condition of homogeneity of variance LSD-t test was used for pairwise comparison; otherwise, Dunnett's T3 method was used. *p* < 0.05 was considered as statistically significant. The Benjamini–Hochberg procedure was applied for multiple testing correction.

## Results

### Overexpression of lncRNA FEZF1-AS1 promotes cellular proliferation and invasion in HOS cells

**Fig. 1:**
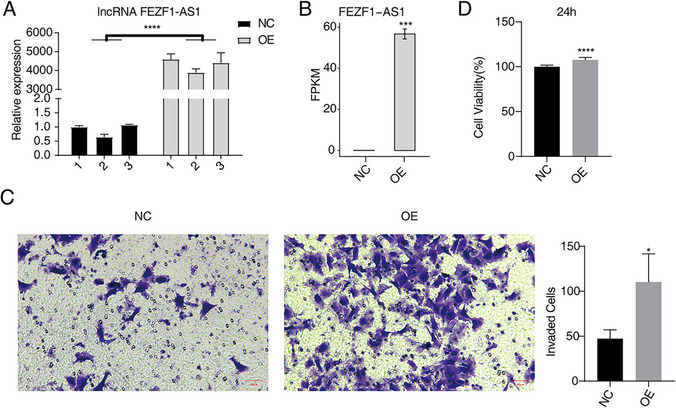
Overexpression (OE) of lncRNA FEZF1-AS1 promotes cellular proliferation and invasion in HOS cells. **A** RT-qPCR results of NC and OE samples. Error bars represent mean ± SEM. *** *p* < 0.001. **B** Bar plot showing the expression pattern and statistical difference of differentially expressed genes (DEGs) for FEZF1-AS1. Error bars represent mean ± SEM. *** *p* < 0.001. **C** Cell invasion results of HOS after OE FEZF1-AS1. **D** Proliferation results of HOS after OE FEZF1-AS1

We successfully established a HOS cell model of OE FEZF1-AS1 gene. A comparison of the experimental group with the control group revealed that FEZF1-AS1 expression was increased by approximately 3893-fold to 4605-fold (*p* < 0.001), which could be utilized in subsequent experiments (Fig. 1A, B). In addition, our studies demonstrated that OE FEZF1-AS1 significantly promoted cell invasion in HOS cells when compared with the control group (Fig. 1C). Furthermore, OE FEZF1-AS1 was found to promote cell proliferation (Fig. 1D).

### FEZF1-AS1 regulates gene expression in HOS cells

**Fig. 2:**
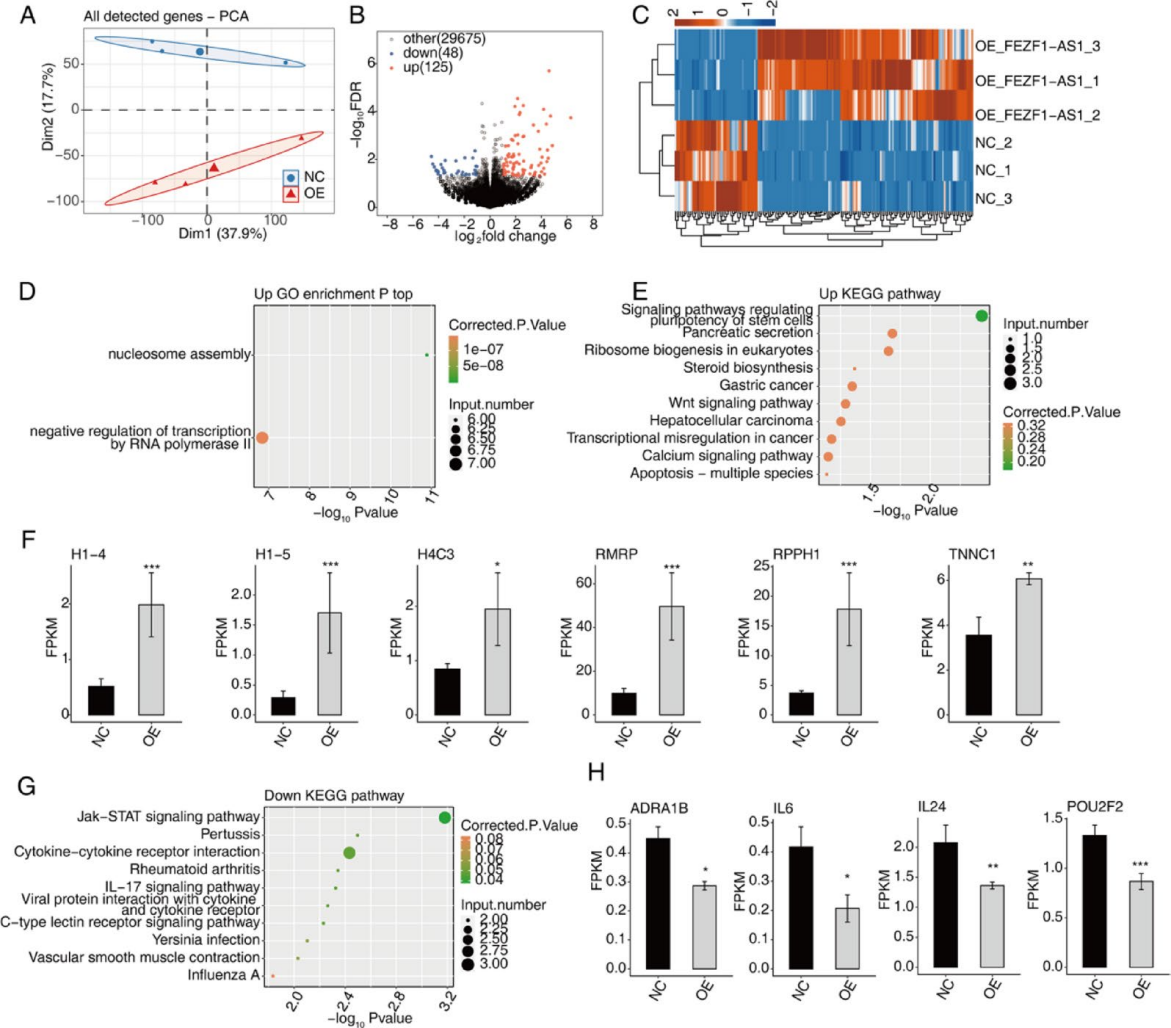
FEZF1-AS1 regulates gene expression in HOS cells. **A** PCA based on FPKM values. **B** Volcano plot of DEGs. **C** Heatmap of DEG expression. **D** GO enrichment for up-regulated DEGs. (E) KEGG enrichment for up-regulated DEGs. **F** Expression patterns of selected DEGs. Error bars: mean ± SEM. *** *p* < 0.001, ** *p* < 0.01, * *p* < 0.05. **G** KEGG enrichment for down-regulated DEGs. **H** Expression patterns of selected DEGs. Error bars: mean ± SEM. *** *p* < 0.001, ** *p* < 0.01, * *p* < 0.05

In order to obtain precise alterations in gene expression and ASEs in OE FEZF1-AS1 cells, high-throughput RNA-seq experiments were selected for the transcriptome analysis of OE FEZF1-AS1 HOS cells. The results of this analysis revealed 173 DEGs in HOS cells of OE FEZF1-AS1, including 125 up-regulated genes and 48 down-regulated genes (Fig. 2A–C). Subsequently, we performed an enrichment analysis. The results of GO enrichment analysis on these up-regulation genes included nucleosome assembly and negative regulation of transcription by RNA polymerase II signaling pathways (Fig. 2D). Furthermore, KEGG results analysis revealed that these genes were enriched in the pluripotency of stem cells, pancreatic secretion, ribosome biogenesis eukaryotic, steroid biosynthesis, gastric cancer, Wnt signaling pathways, hepatocellular carcinoma, transcriptional misregulation in cancer, calcium signaling pathway, and apoptosis-multiple species (Fig. 2E). Subsequently, a statistical analysis was conducted on select enriched genes within these pathways (Fig. 2F), revealing their collective upregulation in OE FEZF1-AS1 HOS cells.Prior studies have demonstrated the pro-cancerous functions of these genes in terms of cancer cell migration, invasion, proliferation, and tumor cell morphology alteration. This further substantiates the credibility of the DEGs that were screened.

Then the results of KEGG enrichment analysis on these 48 down-regulated genes were enriched for Jak-STAT signaling pathway, pertussis, cytokine-cytokine receptor interaction, rheumatoid arthritis, IL-17 signaling pathway, viral proteins interaction with cytokine and cytokine receptor, c-type lectin receptor signaling pathway, *Yersinia* infection, vascular smooth muscle contraction, influenza A, and other signaling pathways (Fig. 2G). The genes enriched in the aforementioned pathways demonstrated significant down-regulation in OE FEZF1-AS1 HOS cells (Fig. 2H). These genes have been documented to impede the proliferation, migration, and invasion of diverse cancer cells, including OS cells, and to suppress the phenotype and tumorigenicity of cancer cells.

### FEZF1-AS1 influences the AS of genes in HOS cells

**Fig. 3:**
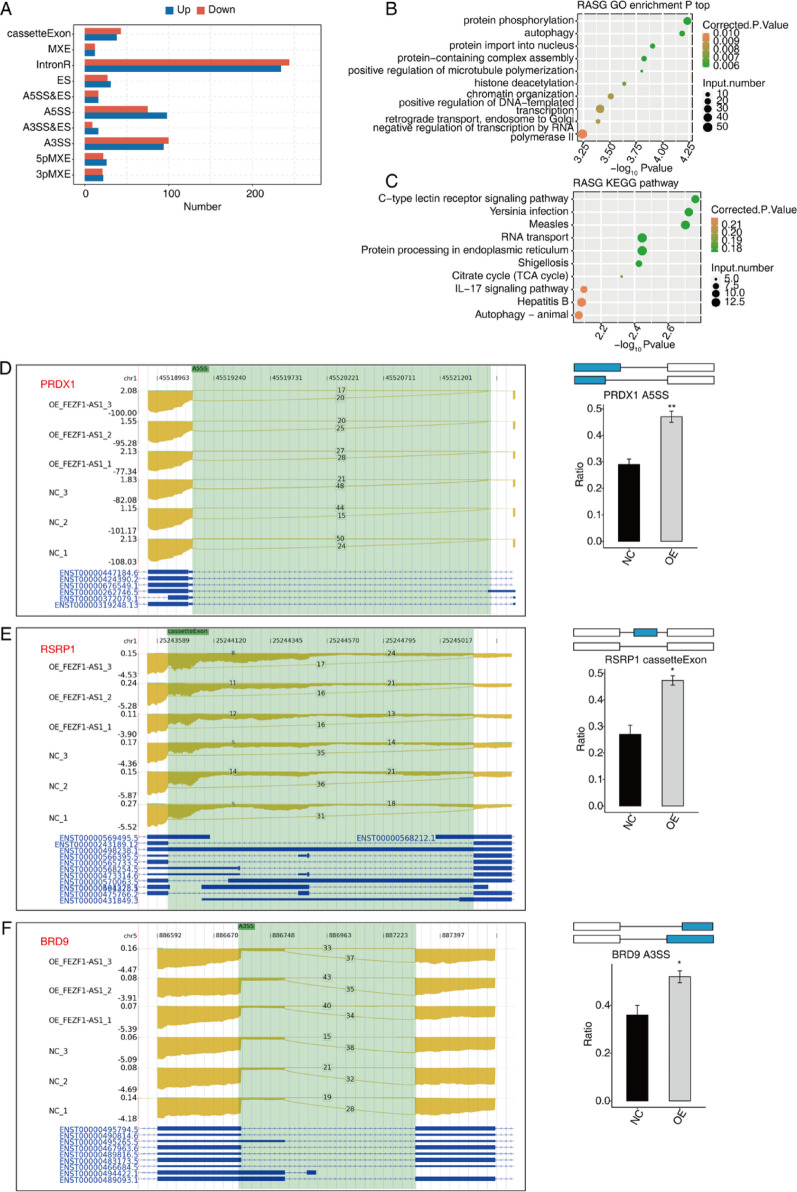
FEZF1-AS1 influences the AS of genes in HOS cells. **A** Bar plot showing the FEZF1-AS1 regulated regulated alternative splicing events (RASEs). **B** GO enrichment for regulated regulated alternative splicing genes (RASGs). **C** KEGG enrichment for regulated RASGs. **D** FEZF1-AS1 regulates AS of PRDX1. Error bars: mean ± SEM. ** *p* < 0.01. **E** FEZF1-AS1 regulates AS of RSRP1. Error bars: mean ± SEM. * *p* < 0.05. **F** FEZF1-AS1 regulates AS of BRD9. Error bars: mean ± SEM. * *p* < 0.05

As shown in Fig. 3A, 1156 significantly different ASEs were affected after OE FEZF1-AS1 (*p* ≤ 0.05). GO enrichment analysis revealed that genes significantly altered by OE FEZF1-AS1 variable splicing were enriched for protein phosphorylation, autophagy, protein import into nucleus, protein-containing complex assembly, positive regulation of microtubule polymerization, histone deacetylation, chromatin organization, positive regulation of DNA-templated, transcription, retrograde transport, endosome to Golgi, and negative regulation of transcription by RNA polymerase II. KEGG results included C-type lectin receptor signaling pathway, *Yersinia* infection, measles, RNA transport, protein processing in endoplasmic reticulum, *Shigella*, TCA cycle, IL-17 signaling pathway, hepatitis B, and autophagy-animal signaling pathway.

In the subsequent phase of the study, nine enriched genes associated with OS in the above pathways were screened, including *PRDX1* (Fig. 3D), *RSRP1* (Fig. 3E), and *BRD9* (Fig. 3F). Furthermore, we identified *PARP6* and *PLD3* (Supplementary Fig. 1), *RAI14*, *NSMCE2*, *YAP1*, and *PGF* (Supplementary Fig. 2) as critical modulators of OS carcinogenesis, as evidenced by their substantial upregulation. Consequently, we postulate that the lncRNA FEZF1-AS1 plays a pivotal role in the regulation of OS development by modulating AS of multiple genes.

## Discussion

One of the most prevalent malignant bone tumors in toddlers and adolescents is OS [34]. The evolution of chemotherapy for OS has progressed from the initial single-agent application to the current multi-agent combination therapy. The combination of multiple chemotherapeutic agents has achieved good therapeutic effects and substantially increased the survival rate of patients. Nevertheless, the prognosis for certain patients remains suboptimal. A substantial body of research has identified the involvement of lncRNAs in the regulation of OS progression, operating through diverse molecular mechanisms [35, 36]. Recent research has demonstrated that the interaction between lncRNA and miRNA plays a significant role in tumourigenesis [37]. For instance, lncRNA–miRNA axes such as MEG3/miR-664a and TUG1/miR-144-3p have been mechanistically linked to OS progression [38, 39]; knockdown of the oncogene lncRNA NEAT1 restores the availability of miR-34c and delays the tumor growth in osteosarcoma [40]. FEZF1-AS1 expression has been reported to be strikingly up-regulated in OS samples and cell lines, suggesting a potential role in the progression of OS [21, 22]. The objective of this study was to identify and briefly describe potential targets of lncRNA FEZF1-AS1 that may affect the levels of transcription and AS in OS cells.

In this study, FEZF1-AS1 was identified as a key factor involved in HOS cell invasion and metastasis. To investigate the specific role and function of FEZF1-AS1 in OS progression, we conducted overexpression of FEZF1-AS1 in HOS cells. The subsequent CCK-8 assay and transwell assay confirmed that enhanced FEZF1-AS1 expression contributed to the proliferation and invasion of HOS cells. Collectively, these findings imply a potential correlation between elevated FEZF1-AS1 expression and the metastatic proclivities of OS.

Furthermore, we leveraged RNA-seq and identified 125 upregulated genes and 48 downregulated genes in overexpressing FEZF1-AS1 HOS cell lines. Through GO analyses and KEGG analyses, we revealed that these genes were enriched in a variety of biosignaling pathways, such as nucleosome assembly and negative regulation of transcription by RNA polymerase II, and apoptosis signaling pathways that are associated with migration, invasion, proliferation, and morphological changes of cancer cells, as well as tumor cell morphology alteration-related pathways. Of particular interest is the validation of the relationship between FEZF1-AS1 and several of these pathways in previous studies on various tumors. For instance, FEZF1-AS1 promoted growth and inhibited apoptosis by regulating miR-363-3p and *PAX6* in retinoblastoma [41]. Moreover, FEZF1-AS1 knockdown significantly inhibited ovarian cancer cell proliferation, and suppressed the apoptosis of ES2 cells [42]. Taken together, these findings suggest that FEZF1-AS1 may contribute to the progression of OS through multiple biological signaling pathways. However, further investigations are necessary to elucidate the specific pathways through which FEZF1-AS1 exerts its effects.

AS is essential for post-transcriptional mRNA processing and plays a key role in many biological processes [43]. We identified 1156 significantly different ASEs between OE FEZF1-AS1 and normal HOS cells. Consistent with previous studies, upon analysis via GO and KEGG, we found that the expression of several genes in these ASEs was significantly associated with OS growth and prognosis. For example, *PRDX1* has been identified as a critical regulator of OS carcinogenesis. Among them, *PRDX1* promotes OS cell proliferation, migration, and metastasis by enhancing phosphorylation of Akt/mTOR [44]. Additionally, *RSRP1* has been shown to play a direct role in the process of AS, engaging in interactions with critical components of the spliceosome. These interactions are believed to contribute to the promotion of the mesenchymal (MES) phenotype characteristic of glioblastoma (GBM) [45]. Overall, our research demonstrates the functional significance of AS events regulated on FEZF1-AS1 in HOS cells across multiple omics levels. Consequently, targeting FEZF1-AS1 emerges as a promising therapeutic approach for OS.

Notwithstanding the insightful findings, this study has several limitations. Firstly, the clinical significance of FEZF1-AS1 remains unexplored, as our validation was restricted to cell line models. Secondly, while bioinformatic predictions suggest FEZF1-AS1-RNA-binding protein (RBP) interactions, direct binding partners were not experimentally verified due to the scope of this transcriptome-focused screening. Thirdly, transcriptomic data require functional validation of candidate molecules like PRDX1/RSRP1. To bridge these gaps, our immediate future work will: (1) Correlate FEZF1-AS1 expression with metastasis/recurrence in OS patient samples; (2) Perform CLIP-seq/RIP-qPCR to map direct RBPs; (3) Establish PDX models for in vivo validation of RASE targets. These steps are essential to advance FEZF1-AS1 toward clinical translation as a potential therapeutic target.

## Conclusions

This study demonstrates the promoting effect of lncRNA FEZF1-AS1 on the proliferation and invasion of HOS cells. Mechanistically, we hypothesize that FEZF1-AS1 may interact with RBPs in HOS cells at the protein level, affecting their localization or expression levels, thereby regulating the splicing of pre-mRNAs and ultimately influencing OS development. Taken together, our findings highlight that FEZF1-AS1 is a major regulator of OS progression and may serve as a potential therapeutic target for OS.

## Supplementary Information

Below is the link to the electronic supplementary material.

Supplementary Material 1

## Data Availability

No datasets were generated or analyzed during the current study.

## Abbreviations

3pMXE: Mutually exclusive 3'UTRs
5pMXE: Mutually exclusive 5'UTRs
A3SS: Alternative 3′ splice site
A5SS: Alternative 5′ splice site
AC: Adjuvant chemotherapy
AS: Alternative splicing
ASE: Alternative splicing event
ceRNA: Competing endogenous RNA
DEG: Differentially expressed gene
ES: Exon skipping
FBS: Fetal bovine serum
FDR: False discovery rate
FEZF1-AS1: FEZ family zinc finger 1-antisense RNA 1
GBM: Glioblastoma
GO: Gene ontology
HOS: Human osteosarcoma
LncRNA: Long non-coding RNA
MES: Mesenchymal
miRNA: MicroRNA
MXE: Mutually exclusive exons
OD: Optical density
OE FEZF1-AS1: Overexpressing lncRNA FEZF1-AS1
OS: Osteosarcoma
RASE: Regulated alternative splicing event
RASG: Regulated alternative splicing gene
RBPs: RNA-binding proteins
RNA-seq: RNA sequencing +
