# Supplementary material for: LncRNA FEZF1-AS1 regulates the gene expression and alternative splicing associated with osteosarcoma
Source: J Orthop Surg Res. 2025 Dec 3;21:24. doi: 10.1186/s13018-025-06357-z (PMC12781780; doi:10.1186/s13018-025-06357-z)
Supplement: Supplementary file 1 — Supplementary Material 1 [file 13018_2025_6357_MOESM1_ESM.zip › supplementary file/Supplementary Figures.doc]

**Figure S1:** FEZF1-AS1 influences the AS of genes in HOS cells.

**Figure S1:** FEZF1-AS1 influences the AS of genes in HOS cells. (A) FEZF1-AS1 regulates AS of PARP6. Left panel: IGV-sashimi plot showing the RASEs and binding sites across mRNA. Reads distribution of RASE is plotted in the up panel and the transcripts of each gene are shown below. Right panel: The schematic diagrams depict the structures of ASEs. RNA-seq validation of ASEs are shown at the bottom of the right panel. Error bars represent mean ± SEM. * *p* < 0.05. (B) FEZF1-AS1 regulates AS of PLD3. Left panel: IGV-sashimi plot showing the RASEs and binding sites across mRNA. Reads distribution of RASE is plotted in the up panel and the transcripts of each gene are shown below. Right panel: The schematic diagrams depict the structures of ASEs. RNA-seq validation of ASEs are shown at the bottom of the right panel. Error bars represent mean ± SEM. * *p* < 0.05.

**Figure S2:** FEZF1-AS1 influences the AS of genes in HOS cells.

**Figure S2:** FEZF1-AS1 influences the AS of genes in HOS cells. (A) FEZF1-AS1 regulates AS of RAI14. Left panel: IGV-sashimi plot showing the RASEs and binding sites across mRNA. Reads distribution of RASE is plotted in the up panel and the transcripts of each gene are shown below. Right panel: The schematic diagrams depict the structures of ASEs. RNA-seq validation of ASEs are shown at the bottom of the right panel. Error bars represent mean ± SEM. * *p* < 0.05. (B) FEZF1-AS1 regulates AS of NSMCE2. Left panel: IGV-sashimi plot showing the RASEs and binding sites across mRNA. Reads distribution of RASE is plotted in the up panel and the transcripts of each gene are shown below. Right panel: The schematic diagrams depict the structures of ASEs. RNA-seq validation of ASEs are shown at the bottom of the right panel. Error bars represent mean ± SEM. * *p* < 0.05. (C) FEZF1-AS1 regulates AS of YAP1. Left panel: IGV-sashimi plot showing the RASEs and binding sites across mRNA. Reads distribution of RASE is plotted in the up panel and the transcripts of each gene are shown below. Right panel: The schematic diagrams depict the structures of ASEs. RNA-seq validation of ASEs are shown at the bottom of the right panel. Error bars represent mean ± SEM. * *p* < 0.05. (D) FEZF1-AS1 regulates AS of PGF. Left panel: IGV-sashimi plot showing the RASEs and binding sites across mRNA. Reads distribution of RASE is plotted in the up panel and the transcripts of each gene are shown below. Right panel: The schematic diagrams depict the structures of ASEs. RNA-seq validation of ASEs are shown at the bottom of the right panel. Error bars represent mean ± SEM. ** *p* < 0.01.
